# Tear-Based Oxidative Stress Biomarkers in Primary and Sarcoidosis-Associated Dry Eye Disease

**DOI:** 10.3390/ijms27042071

**Published:** 2026-02-23

**Authors:** Calina-Anda Sandu, Vlad Constantin Donica, Ioana-Miruna Balmus, Ioana Madalina Bilha, Cosmin Victor Ganea, Ioana Alexandra Sandu, Anisia Iuliana Alexa, Alexandra Lori Donica, Valentina Esanu, Alin Ciobica, Camelia Margareta Bogdanici

**Affiliations:** 1 Grigore T. Popa University of Medicine and Pharmacy, 7000115 Iasi, Romania; calina-anda.sandu@umfiasi.ro (C.-A.S.); madalinabilha@gmail.com (I.M.B.); cosmin-victor.ganea@umfiasi.ro (C.V.G.); anisia-iuliana.alexa@umfiasi.ro (A.I.A.); costachescu.alexandra-lori@d.umfiasi.ro (A.L.D.); camelia.bogdanici@umfiasi.ro (C.M.B.); 2 Department of Exact Sciences and Natural Sciences, Institute of Interdisciplinary Research, “Alexandru Ioan Cuza” University, 700057 Iasi, Romania; balmus.ioanamiruna@yahoo.com; 3 CENEMED Platform for Interdisciplinary Research, University of Medicine and Pharmacy “Grigore T. Popa”, 7000115 Iasi, Romania; alin.ciobica@uaic.ro; 4 Arcadia Medical Rehabilitation Hospital, 707035 Barnova, Romania; ioana-alexandra.sandu@umfiasi.ro; 5 Pneumonological Hospital, 700115 Iasi, Romania; esanuv@gmail.com; 6 Department of Biology, “Alexandru Ioan Cuza” University of Iasi, 700505 Iasi, Romania; 7 “Ioan Haulica” Institute, Apollonia University, 700511 Iasi, Romania; 8 “Olga Necrasov” Center, Biomedical Research Group, Romanian Academy, 700481 Iasi, Romania

**Keywords:** sarcoidosis, tear film alterations, oxidative stress, dry eye disease, ocular surface inflammation, antioxidant imbalance

## Abstract

Dry eye disease (DED) has increasingly been linked to oxidative stress; however, the specific redox mechanisms underlying different clinical phenotypes remain incompletely understood. This study aimed to evaluate tear film oxidative stress profiles in patients with primary DED and sarcoidosis-associated DED (S-DED) by assessing lipid peroxidation, antioxidant enzyme activity, and total tear protein content, and to explore their relationship with clinical tear film dysfunction. Tear samples were analyzed for superoxide dismutase (SOD) and glutathione peroxidase (GPx) activities, as well as for malondialdehyde (MDA) and total protein levels, alongside standard clinical tests of tear film stability and secretion. Both DED groups exhibited significant oxidative alterations compared to controls, but with distinct redox signatures. Primary DED was characterized by markedly increased tear MDA levels, indicating predominant lipid peroxidation, whereas S-DED showed a more pronounced impairment of antioxidant defense, reflected by preserved or increased SOD activity in the context of significantly reduced GPx activity. Total tear protein levels were reduced in both groups, with evidence suggesting qualitative protein alterations in S-DED. The tear collection method significantly influenced the measured levels of several oxidative stress markers, underscoring the importance of sampling technique when interpreting tear-based redox profiles. Oxidative stress markers correlated with clinical measures of tear film dysfunction, supporting their physiological relevance. These findings demonstrate that DED encompasses heterogeneous oxidative stress mechanisms and that sarcoidosis acts as a modifier of ocular surface redox homeostasis. Distinct tear-based redox profiles differentiate primary from sarcoidosis-associated dry eye, highlighting the potential value of oxidative biomarkers for phenotyping DED beyond tear deficiency alone.

## 1. Introduction

Oxidative stress refers to a pathophysiological imbalance between the generation of reactive oxygen species (ROS) and the capacity of antioxidant defense systems to neutralize them [1,2]. Although oxidative metabolism primarily takes place in mitochondria and the endoplasmic reticulum, disruption of redox homeostasis represents more than the accumulation of metabolic byproducts, reflecting a fundamental alteration in cellular signaling and regulatory processes [2,3]. At physiological concentrations, ROS act as essential signaling molecules involved in cell proliferation, immune responses, and regulation of gene expression [1,2]. Mitochondria are the main endogenous source of ROS, with approximately 90% generated as byproducts of the electron transport chain [2,3]. In parallel, the ocular surface is exposed to exogenous oxidative stressors, including ultraviolet radiation and environmental pollutants, which further enhance free radical production by impairing protective enzymatic mechanisms [2].

The antioxidant defense system is largely regulated by the transcription factor Nrf2 and includes both enzymatic and non-enzymatic components [1,2]. Key enzymatic antioxidants include superoxide dismutase (SOD) and glutathione peroxidase (GPx), which play critical roles in limiting ROS-mediated damage. When ROS generation exceeds the buffering capacity of these defenses, oxidative injury to cellular macromolecules occurs. Polyunsaturated fatty acids within cell membranes are particularly vulnerable, and their peroxidation leads to the formation of reactive aldehydes such as malondialdehyde (MDA) [2]. These oxidative processes directly impact membrane integrity and protein function, contributing to tear film instability and ocular surface dysfunction.

Although oxidative stress has been implicated in the pathogenesis of dry eye disease (DED), most available studies have evaluated oxidative markers in DED as a single entity. Comparative data addressing whether distinct systemic inflammatory conditions, such as sarcoidosis, modify the tear-based redox profile are scarce. Consequently, it remains unclear whether sarcoidosis-associated dry eye (S-DED) represents merely a quantitative form of tear deficiency or a qualitatively distinct oxidative phenotype.

Within this context of heterogeneous oxidative mechanism, sarcoidosis represents a complex multisystem inflammatory disease of unknown etiology, histopathologically defined by non-caseating granulomas and a marked predilection for pulmonary, lymphatic, and ocular involvement [4]. Ocular sarcoidosis accounts for the third most frequent clinical manifestation of the disease and affects approximately 30–60% of patients [5,6]. Due to its heterogeneous presentation and capacity to involve multiple organ systems, sarcoidosis has been described as a “promiscuous disease” [7].

Ocular surface involvement is an underrecognized but clinically relevant manifestation of sarcoidosis. Granulomatous infiltration of the lacrimal and meibomian glands is closely associated with the development of DED, manifested by reduced Schirmer I test values and shortened tear film breakup time (BUT) [8,9]. However, increasing evidence indicates that dry eye associated with sarcoidosis is not defined solely by reduced tear secretion, but also by qualitative alterations in tear film composition and stability, including abnormal tear film patterns and glandular dysfunction, even in the absence of severe aqueous deficiency. These observations suggest that ocular surface dysfunction in sarcoidosis may reflect underlying biochemical and molecular disturbances rather than tear deficiency alone [10].

Reported mean Schirmer I values of 12.9 ± 10.5 mm in patients with ocular sarcoidosis, compared with 24.3 ± 10.5 mm in healthy controls, suggest concomitant meibomian gland atrophy and gland dropout [8,9]. This dysfunction predominantly affects the main lacrimal gland, leading to impairment of the neural reflex arc, while accessory lacrimal secretion remains relatively preserved [8]. Despite these well-documented clinical findings, the molecular mechanisms underlying ocular surface damage in sarcoidosis, particularly those related to oxidative stress, remain insufficiently characterized.

Therefore, the aim of this study was to evaluate tear-based oxidative stress markers, including MDA, SOD, GPx, and total tear protein content, in patients with primary DED and S-DED, and to explore their relationship with clinical parameters of tear film dysfunction. By comparing these two clinical phenotypes, this study seeks to determine whether sarcoidosis acts as a modifier of ocular surface redox homeostasis and contributes to distinct oxidative stress signatures in dry eye disease.

## 2. Results

We analyzed 60 eyes from 30 patients divided into Control, S-DED, and DED groups based on the method of acquisition. Descriptive parameters regarding median (interquartile) values can be found in Table 1.

**Figure 1:**
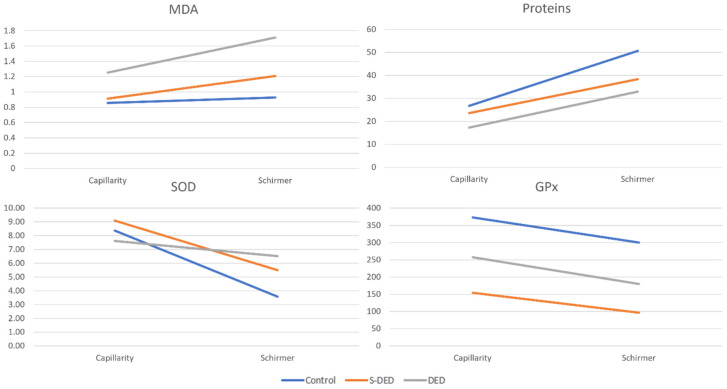
Between groups analysis of MDA, Proteins, SOD and GPx based on different sampling methods.

Linear mixed model analysis provided data regarding estimated marginal means (Table 2), pairwise comparisons (Table 3) and adjusted means for each group (Table 4), with differences based on sampling method and analyzed group for MDA, Proteins, SOD and GPx being highlighted in Figure 1. Mann–Whitney group comparison was performed in Table 5.

### 2.1. BUT

**Figure 2:**
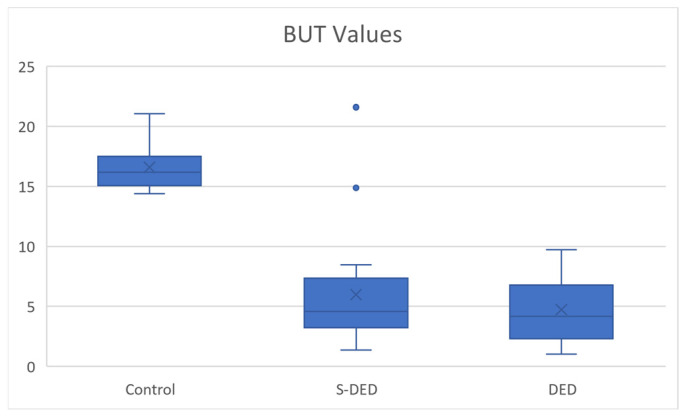
BUT values across studied groups.

Break-up time values differed significantly between groups, with the highest values observed in the control group, followed by the S-DED group, and the lowest values in the DED group (Figure 2) No significant inter-eye differences were identified (paired comparison, *p* = 0.643); therefore, data from both eyes were pooled for analysis. The median BUT was 16.18 in controls, compared with 4.57 in the S-DED group and 4.17 in the DED group. Group comparisons using the Mann–Whitney *U* test demonstrated significant differences between controls and S-DED and between controls and DED (*p* < 0.001), while no statistically significant difference was observed between the S-DED and DED groups (*p* > 0.05) (Table 5).

### 2.2. Schirmer Test Values

**Figure 3:**
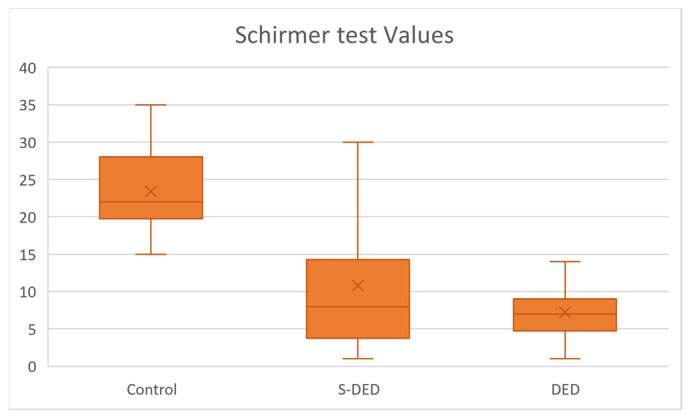
Schirmer test values across studied groups.

Schirmer test values showed a similar pattern to BUT, with highest values in the control group, intermediate values in the S-DED group, and lowest values in the DED group (Figure 3). No significant inter-eye differences were detected (paired comparison, *p* = 0.736), and measurements from both eyes were therefore pooled. The median Schirmer value was 22 in controls, compared with 8 in the S-DED group and 7 in the DED group. Mann–Whitney *U* testing revealed significant differences between controls and S-DED and between controls and DED (both *p* < 0.001), whereas no significant difference was found between the S-DED and DED groups (*p* > 0.05) (Table 5).

### 2.3. SOD

Linear mixed-effects modeling demonstrated a significant main effect of tear collection method on SOD activity (*p* = 0.002), while neither the patient group effect (*p* = 0.509) nor the Group × Collection Method interaction (*p* = 0.304) reached statistical significance. These findings indicate that SOD activity is primarily influenced by the tear collection technique and not by disease status.

When averaged across collection methods, adjusted SOD activity did not differ significantly between groups, with mean values of 5.97 ± 0.75 in controls, 7.29 ± 0.75 in S-DED, and 7.07 ± 0.75 in DED. Pairwise comparisons confirmed the absence of statistically significant differences between any of the groups.

Across all patient groups, capillary-collected samples exhibited significantly higher adjusted SOD activity (8.36 ± 0.71) compared with Schirmer-collected samples (5.20 ± 0.71, *p* = 0.002), indicating a strong and consistent method-related effect.

Because no significant interaction was observed, the effect of collection method was consistent across all groups. In each group, capillary-collected samples yielded higher SOD activity than Schirmer-collected samples, while the relative similarity between patient groups was preserved regardless of tear collection technique.

### 2.4. GPx

Linear mixed-effects modeling demonstrated a significant main effect of patient group on GPx activity (*p* = 0.002), while neither the tear collection method (*p* = 0.144) nor the Group × Collection Method interaction (*p* = 0.985) reached statistical significance. These findings indicate that GPx activity differs according to disease status but is not significantly influenced by the tear collection technique.

When averaged across collection methods, adjusted GPx activity was highest in controls (337.6 ± 40.8), lowest in S-DED (125.4 ± 40.8), and intermediate in DED (218.6 ± 40.8). Pairwise comparisons revealed a statistically significant reduction in GPx activity in S-DED compared with controls (212.22 ± 56.73, *p* = 0.001). Differences between DED and controls (*p* = 0.122), as well as between S-DED and DED (*p* = 0.318), did not reach statistical significance.

Across all patient groups, adjusted GPx activity did not differ significantly between capillary-collected samples (261.52 ± 32.75) and Schirmer-collected samples (192.93 ± 32.75, *p* = 0.144), indicating that GPx measurements are not sensitive to the method of tear collection.

In the absence of a significant interaction, the pattern of group differences in GPx activity was preserved across both collection methods. Within each group, capillary-collected samples tended to show numerically higher GPx activity than Schirmer-collected samples; however, these differences were not statistically significant.

### 2.5. MDA

Linear mixed-effects analysis revealed significant effects of patient group (*p* < 0.001), tear collection method (*p* < 0.001), and a significant Group × Collection Method interaction (*p* = 0.035) for MDA indicating that the effect of tear collection technique differed according to disease status.

Estimated marginal means averaged across collection methods showed progressively higher MDA levels from controls (0.89 ± 0.05) to S-DED (1.06 ± 0.05) and DED (1.48 ± 0.05). Pairwise comparisons demonstrated significantly higher MDA levels in DED compared with both controls (0.59 ± 0.07, *p* < 0.001) and S-DED (0.42 ± 0.07, *p* < 0.001), while the difference between controls and S-DED did not reach statistical significance (*p* = 0.076). Across all groups, Schirmer-collected samples showed higher adjusted MDA levels (1.28 ± 0.04) compared with capillary-collected samples (1.01 ± 0.04, *p* < 0.001).

Due to the significant interaction, comparisons between collection methods were examined within each group. No significant difference between capillary and Schirmer sampling was observed in controls (0.86 ± 0.07 vs. 0.93 ± 0.07; *p* = 0.331). In contrast, Schirmer-collected samples yielded significantly higher MDA levels than capillary-collected samples in S-DED (1.21 ± 0.07 vs. 0.91 ± 0.07; *p* < 0.001) and in DED (1.71 ± 0.07 vs. 1.25 ± 0.07; *p* < 0.001), with the largest method-related difference observed in the DED group.

### 2.6. Total Proteins

Linear mixed-effects modeling demonstrated significant main effects of patient group (*p* < 0.001) and tear collection method (*p* < 0.001) on total tear protein concentrations, while the Group × Collection Method interaction was not statistically significant (*p* = 0.251), indicating that the effect of tear collection technique was consistent across groups.

Estimated marginal means averaged across collection methods showed the highest protein concentrations in controls (38.74 ± 2.2), followed by S-DED (30.96 ± 2.2) and DED (25.11 ± 2.2). Pairwise comparisons revealed significantly lower levels in DED compared with controls (13.63 ± 3.02, *p* < 0.001). S-DED also showed lower protein levels than controls (7.78 ± 3.02, *p* = 0.039), while the difference between S-DED and DED did not reach statistical significance (*p* = 0.174).

Across all groups, Schirmer-collected samples yielded significantly higher protein concentrations than capillary-collected samples (*p* < 0.001). Because protein values were expressed as concentration (mg/mL), the higher levels observed in Schirmer-collected samples likely reflect differences in tear composition and strip-related protein mobilization rather than simple volumetric dilution.

When examined within each group, Schirmer sampling consistently resulted in higher protein levels compared with capillary sampling in controls, S-DED, and DED (all *p* < 0.001), with similar levels of differences between groups.

### 2.7. Correlations

Subgroup Spearman correlations for the capillary sampling method in the control group found a strong positive correlation between BUT and Schirmer values (r = 0.823, *p* = 0.003). The S-DED group presented a strong positive correlation between MDA and SOD (r = 0.723, *p* = 0.018). In the DED group we found a moderate negative correlation between SOD and Schirmer test values (r = −0.646, *p* = 0.043) and strong positive between BUT and Schirmer test values (r = 0.848, *p* = 0.002).

The Schirmer Sampling method found the control group had a statistically significant strong positive correlation between BUT and Schirmer values (r = 0.775, *p* = 0.008). The S-DED group had a statistically significant strong positive correlation between MDA and BUT (r = 0.673, *p* = 0.033), MDA and Schirmer Test (r = 0.79, *p* = 0.007), and between BUT and Schirmer test values (r = 0.839, *p* = 0.002). In the DED group there was a strong positive correlation between Schirmer and BUT test values (r = 0.908, *p* < 0.001).

## 3. Discussion

### 3.1. Oxidative Stress in Dry Eye Disease: General Considerations

DED has increasingly been recognized as a disorder driven in large part by oxidative stress and an inability of the ocular surface to maintain redox homeostasis. Oxidative damage in the lacrimal functional unit can manifest as tissue atrophy and fibrosis, inflammatory cell infiltration, and accelerated lipid and DNA oxidation with ensuing cell apoptosis—pathological changes that ultimately lead to the development of DED [11]. In a healthy eye, the tear film itself serves as a critical antioxidant defense barrier, containing enzymes such as SOD and GPx that neutralize ROS and protect the ocular surface [12]. This complex tear film—comprising lipid, aqueous, and mucous layers—not only lubricates and nourishes the cornea but also guards against oxidative damage and environmental stress. When tear film stability is compromised (for example, by deficient mucins or increased evaporation), the resulting hyperosmolarity triggers a cascade of oxidative stress at the ocular surface. Experimental models of DED have shown that hyperosmolar conditions provoke a surge in ROS production in corneal epithelial cells while concurrently downregulating antioxidant defenses like SOD2 and GPx, thereby disrupting redox balance [13]. This creates a vicious cycle: tear hyperosmolarity and inflammation generate ROS that overwhelm local antioxidants, leading to lipid peroxidation of cell membranes and protein oxidation in tears. Over time, this persistent oxidative environment directly damages the corneal epithelium and conjunctiva, instigates chronic inflammation, and exacerbates tear film instability—all hallmark features of DED [11,13]. Therefore, assessing oxidative stress through biomarkers in tear fluid has strong pathophysiologic rationale in DED. Key biomarkers of interest include MDA, a stable end-product of lipid peroxidation, and the activities of antioxidant enzymes like SOD and GPx, alongside total tear protein levels which reflect the functional integrity of the tear film [11,12,13,14].

### 3.2. Lipid Peroxidation and Elevated MDA in DED

A significant increase in tear MDA levels was observed in patients with DED, encompassing both primary and sarcoidosis-associated forms, compared to healthy controls, consistent with enhanced lipid peroxidation at the ocular surface. This result is in line with numerous reports identifying MDA as a central marker of oxidative stress in dry eye. MDA is a reactive aldehyde generated by ROS-driven degradation of polyunsaturated fatty acids in cell membranes, and its accumulation reflects oxidative damage to cellular lipids [11]. Elevated MDA has been consistently detected in the tears and conjunctival tissues of DED patients, often correlating with the severity of tear dysfunction and ocular surface damage [11,15]. Meta-analytic evidence confirms that tear MDA is significantly higher in DED patients than in healthy individuals, making it one of the most reproducible oxidative stress markers in ocular surface disease [15]. This increase in MDA directly signifies membrane lipid peroxidation, which contributes to tear film instability and can amplify the self-perpetuating cycle of ocular surface inflammation, epithelial injury, and tear hyperosmolarity [14,15]. Indeed, increased MDA in tears has been linked to clinical indicators of dry eye severity—for example, higher MDA is associated with shorter tear film break-up time, reduced Schirmer values, greater ocular surface staining, and even goblet cell loss in conjunctiva according to prior studies [16]. Our findings concur with these associations: patients with higher tear MDA exhibited worse tear film stability and secretion, underscoring that lipid peroxidation is not merely a biochemical observation but a driver of clinical dysfunction [16,17]. It is noteworthy that MDA (and a related peroxidation byproduct 4-hydroxynonenal) have been rated as high-evidence markers of ocular surface oxidative stress, given their consistent presence in DED and strong correlations with clinical parameters [16]. By serving as a stable end-product of lipid oxidation, MDA effectively integrates the upstream oxidative events and provides a measurable indicator of ongoing oxidative damage in DED tears [15,18]. The presence of increased tear MDA levels supports the concept that DED is characterized by active lipid peroxidation at the ocular surface, thereby linking molecular oxidative damage to tear film instability and clinical manifestations [11,14,15].

Beyond DED, elevated tear MDA has been implicated as a marker of oxidative stress in other ocular pathologies, supporting its broader relevance at the ocular surface. In patients with central serous chorioretinopathy (CSCR), significantly higher tear MDA levels were reported in the acute form compared to chronic active disease, and tear MDA concentrations correlated with the height of serous retinal detachment assessed by spectral-domain optical coherence tomography. These findings suggest that tear MDA reflects not only local lipid peroxidation but also disease activity and severity within the eye. Collectively, such observations reinforce the concept that malondialdehyde represents a sensitive and biologically meaningful tear-based biomarker of oxidative stress across distinct ocular diseases, highlighting the value of optimized analytical approaches for its accurate quantification [18].

### 3.3. Antioxidant Enzymes SOD and GPx: An Imbalanced Defense

In parallel with enhanced oxidative damage, our DED patients demonstrated a marked imbalance in their tear antioxidant enzyme activities. An increase (or preservation) of SOD activity was observed alongside a significant decrease in GPx activity, particularly pronounced in the sarcoidosis-associated DED group. This dissociation between SOD and GPx is a signature of an impaired antioxidant defense system at the ocular surface. SOD is the first-line enzyme that converts superoxide radicals into hydrogen peroxide [1,15]. In our patients, tear SOD was elevated relative to controls, suggesting a compensatory upregulation in response to excess superoxide generation. Not all studies have reported uniform changes in SOD for dry eye, but a pattern emerges: many DED cases (especially non-Sjögren DED) exhibit local increases in SOD activity as the system attempts to counteract ROS, whereas in some other ocular conditions SOD can be reduced if antioxidant reserves are exhausted [15,19,20]. Importantly, an increase in SOD does not necessarily equate to effective protection—rather, it can indicate an ongoing oxidative burden that triggers SOD as a compensatory but insufficient response [15,21]. Our findings support this interpretation: despite higher SOD, patients did not restore redox homeostasis because the downstream detoxification of peroxides was compromised. Specifically, tear GPx activity was significantly reduced in DED, with the most pronounced drop in the S-DED subgroup. GPx (a glutathione-dependent peroxidase) is essential for neutralizing hydrogen peroxide and organic peroxides generated after SOD activity; a deficit in GPx means that hydrogen peroxide can accumulate and convert into more reactive radicals, propagating lipid and protein oxidation [15,22]. Indeed, previous investigations into chronic oxidative stress conditions have noted this phenomenon of “imbalanced” antioxidant response: for example, in fibrotic orbital disease, cells showed increased SOD activity coupled with decreased GPx, leading to excess hydrogen peroxide and oxidative damage [22]. The tear data obtained in the present analysis mirror the paradigm of an incomplete antioxidant cascade, with SOD-driven peroxide generation occurring in the absence of sufficient GPx-mediated detoxification [23,24]. This imbalance aligns with recent conceptualizations framing DED as a disease of impaired redox homeostasis rather than just excess oxidants [24]. In other words, it is the failure to maintain a functional balance between superoxide dismutation and peroxide detoxification that sustains oxidative stress in DED [24]. Elevated SOD levels likely reflect an active oxidative milieu and are consistent with reports identifying SOD as a dominant tear film antioxidant that can be upregulated in DED, particularly in milder or non-Sjögren forms [15]. However, the concomitant depression of GPx activity reveals a breakdown in the antioxidant defense network—a finding also noted in systemic inflammatory contexts. For instance, sarcoidosis and other chronic inflammatory diseases are characterized by disorganized antioxidant responses: SOD may be upregulated in certain compartments, but GPx and other glutathione-dependent systems become depleted or functionally insufficient [25]. These findings provide evidence of a similar tear film antioxidant failure in DED. Rather than a coordinated adaptive upregulation of all antioxidant defenses, a skewed response is observed that ultimately favors the persistence of ROS byproducts. Notably, this is supported by other DED studies where total antioxidant capacity is overwhelmed; some have even found SOD activity eventually declining when defenses are exhausted in chronic disease, despite an initial rise [26]. Taken together, the simultaneous increase in SOD (as a compensatory mechanism) and decrease in GPx (as a sign of antioxidant exhaustion) in our patients illustrates a tear film environment under oxidative duress and unable to effectively neutralize intermediate peroxides [15,22,24]. This dysfunctional antioxidant profile likely contributes to continuous oxidative damage to the ocular surface, fueling inflammation and tissue injury in dry eye [21,24].

### 3.4. Tear Protein Alterations Under Oxidative Stress

Another significant finding is the reduction in total tear protein concentration in patients with DED compared to controls. Both primary DED and S-DED subjects showed lower total protein levels in tears, with the largest decrease observed in primary DED. This observation is consistent with reports that chronic dry eye is accompanied by qualitative and quantitative changes in the tear proteome [27]. The tear film contains numerous proteins (enzymes, growth factors, immunoglobulins, mucins, etc.) that are vital for ocular surface lubrication, antimicrobial defense, epithelial integrity, and antioxidant protection. In DED, loss of these protective tear proteins is a common feature, reflecting both reduced secretion by a dysfunctional lacrimal gland and increased protein degradation in an inflamed, oxidative environment [15,27]. Prior studies have noted that patients with DED generally have lower total tear protein content than healthy individuals, and this decrease correlates with tear film instability and glandular dysfunction [15]. Our results reinforce that concept; the diminished tear proteins likely indicate that the lacrimal gland’s secretory function is impaired—possibly due to inflammatory damage—and that key protective proteins (such as lactoferrin, lipocalin, and tear-specific prealbumin) are either not produced in sufficient quantity or are being consumed by oxidative reactions on the ocular surface [15,27]. Oxidative stress can directly modify tear proteins via protein carbonylation and other post-translational modifications, leading to dysfunctional proteins that may precipitate out or be cleared from the tear film [14,21]. Accordingly, protein concentrations measured in Schirmer-collected tears should be interpreted as reflecting altered tear composition under reflex and inflammatory conditions rather than simple dilution effects. In an oxidative microenvironment, crucial tear components (for example, antioxidant proteins like lactoferrin, lysozyme, or peroxidases) can be inactivated or degraded, exacerbating tear film instability and reducing the eye’s natural defenses [15]. The greater reduction in total protein we observed in primary DED (compared to S-DED) might suggest a more severe lacrimal gland impairment in idiopathic dry eye, whereas in S-DED the protein changes could be more qualitative (oxidation-related) than purely quantitative. This interpretation is supported by the observation that in S-DED, despite slightly higher total tear protein levels compared to primary DED, a greater proportion of oxidized or dysfunctional proteins may contribute to disease pathogenesis. It is also noteworthy that when analyzing tear antioxidants, some authors recommend normalizing enzyme activities to total protein content to account for variations in tear fluid volume and protein loss [27]. In our study, the robust drop in GPx activity remained evident regardless of collection method or normalization, highlighting GPx as a reliable marker of antioxidant status. Nonetheless, the decline in total tear proteins itself serves as an indirect indicator of ocular surface health—it points to a disrupted secretory function and a tear film under oxidative/inflammatory stress [15,27]. Overall, the tear protein depletion in DED complements the evidence of redox imbalance: as oxidative stress and inflammation persist, they compromise the tear film’s protein composition and further diminish its protective capabilities [15,21,27].

### 3.5. Distinct Redox Profiles in Primary vs. Sarcoidosis-Associated DED

A novel aspect of this analysis is the comparison between primary DED and S-DED, which revealed notable differences in oxidative stress profiles. While both groups exhibited a departure from physiological redox homeostasis, the nature of the imbalance differed between primary and secondary dry eye. Primary DED patients showed a more pronounced increase in tear MDA levels, suggesting that lipid peroxidation represents a dominant feature of oxidative injury in idiopathic dry eye. In contrast, S-DED patients, who present an underlying systemic inflammatory condition, exhibited a comparatively attenuated MDA elevation but a more severe disruption of antioxidant enzyme activity, most notably a marked reduction in GPx alongside preserved or increased SOD activity.

This dissociation between antioxidant enzyme systems aligns with accumulating evidence that sarcoidosis is characterized by persistent immune-mediated ROS production and an exhausted or disorganized antioxidant response at the systemic level. Activated macrophages in sarcoidosis generate increased amounts of superoxide in an interferon-γ–dependent manner, contributing to a sustained oxidative burden even during clinically stable disease [28]. In parallel, several studies have documented diminished systemic antioxidant capacity in sarcoidosis, including reduced total antioxidant status, depletion of glutathione-dependent defenses, and impaired activity of key enzymatic antioxidants [29]. Such a redox environment may favor chronic oxidative stress without necessarily leading to extreme accumulation of terminal lipid peroxidation products and has been described as diffuse and sustained across multiple biological compartments [25,30].

Accordingly, the tear film phenotype observed in S-DED may reflect the extension of this systemic redox imbalance to the ocular surface. The significantly reduced GPx activity suggests an impaired capacity to neutralize hydrogen peroxide and organic peroxides generated downstream of SOD activity, rendering preserved or increased SOD function insufficient to prevent ongoing oxidative damage [24,25]. In contrast, oxidative stress in primary DED may manifest predominantly as episodic lipid peroxidation bursts within an unstable tear film, reflected by markedly elevated MDA levels and driven by tear film break-up and surface exposure. Together, these findings highlight that while both primary and secondary DED share a common theme of redox imbalance, sarcoidosis acts as a modifier of ocular surface redox homeostasis by shaping the qualitative nature of oxidative injury. Sarcoidosis-associated dry eye thus appears to be defined more by a breakdown in the antioxidant cascade—particularly the SOD–GPx axis—than by excessive lipid peroxidation alone, reinforcing the concept that DED is a heterogeneous condition requiring subtype-specific mechanistic consideration [24,25].

### 3.6. Oxidative Stress Markers and Tear Film Functional Impairment

The strong link between oxidative stress and tear film dysfunction in DED is further supported by correlations identified in the present cohort and in previous clinical studies. In the present analysis, higher levels of oxidative stress markers were associated with poorer tear film performance; specifically, increased tear MDA concentrations were associated with shorter tear break-up time and lower Schirmer test values, indicating more pronounced tear instability and reduced tear secretion. These observations are consistent with reports showing that MDA and other lipid peroxidation markers correlate inversely with tear film stability and production [16]. Collectively, these data indicate that increasing oxidative damage within the tear film is accompanied by a progressive impairment of its ability to maintain a stable and continuous ocular surface. In addition, oxidative stress and inflammation are known to disrupt ocular surface neural feedback mechanisms and lacrimal gland function, thereby reducing reflex tear secretion and mucin production, effects that are reflected in standard clinical test results. The associations observed in the present cohort, together with prior reports, support the relevance of tear BUT and Schirmer testing as functional correlates of biochemical oxidative stress at the ocular surface [16,17]. Moreover, oxidative modifications of tear components directly compromise tear film quality: oxidized lipids destabilize the lipid layer, while oxidized or depleted proteins impair tear spreading and wettability, promoting faster evaporation and film break-up [15,17]. A bidirectional relationship further amplifies this process, as tear film dysfunction and hyperosmolarity can in turn enhance ROS generation, establishing a self-perpetuating cycle of oxidative injury [13]. Experimental evidence supports the functional relevance of this redox imbalance, as enhancement of tear antioxidant defenses has been shown to improve tear film stability. For instance, upregulation of SOD activity through antioxidant intervention resulted in significant improvement of tear break-up time despite minimal effects on tear volume [31], suggesting that oxidative damage primarily affects tear film quality at early stages and may be partially reversible. Similarly, treatment with a mitochondrial-targeted antioxidant normalized tear SOD and GPx activity, reduced MDA accumulation, and attenuated inflammatory signaling, leading to improvement of the tear film profile in experimental models [26]. In line with these observations, the present findings provide a mechanistic framework linking clinical signs of DED to a persistent oxidative environment in tears, characterized by elevated MDA and an imbalanced SOD/GPx system, which together promote tear film instability and epithelial damage [11,17]. This supports the broader concept that oxidative stress acts both as a consequence and a driver of tear film dysfunction, positioning redox biomarkers as indicators of disease severity and potential targets for therapeutic modulation [15,31].

A similar pattern of redox imbalance has been described in other ocular surface and corneal disorders, such as keratoconus, further supporting the pathological relevance of an uncoordinated antioxidant response. In keratoconus, oxidative stress is characterized not by a uniform depletion of antioxidant defenses, but by enzymatic imbalance within the antioxidant cascade, with altered SOD activity insufficiently counterbalanced by downstream peroxide-detoxifying mechanisms. This dysregulation has been linked to increased oxidative damage, extracellular matrix degradation, and apoptotic signaling within corneal tissue [32,33]. The parallels between keratoconus and the oxidative profile observed in dry eye disease, particularly in sarcoidosis-associated DED, reinforce the concept that disruption of coordinated antioxidant defense, rather than absolute ROS excess alone, represents a critical driver of ocular surface pathology.

Taken together, these findings indicate that oxidative stress in dry eye disease is closely linked to functional impairment of the tear film, as reflected by reduced tear break-up time and Schirmer values. However, in the presence of systemic inflammatory disease such as sarcoidosis, tear dysfunction may be driven predominantly by qualitative alterations in tear composition rather than by reduced tear volume alone. In this context, diagnostic approaches that assess tear film structure and physicochemical properties become particularly relevant. It has been shown that the tear ferning test can better discriminate sarcoidosis-associated dry eye from primary DED and healthy controls by revealing characteristic crystallization patterns indicative of altered tear composition and osmolarity. These observations support the concept that qualitative functional tests may provide added diagnostic value in S-DED, complementing conventional quantitative assessments [34].

### 3.7. Redox Imbalance: A Central Mechanism in DED

Collectively, evidence from the present analysis and the existing literature suggests that dry eye disease can be fundamentally characterized as a disorder of redox imbalance at the ocular surface [14,15]. The concurrent alterations identified across tear oxidative stress markers delineate a coherent profile of the tear film milieu in DED. In summary, DED tears are characterized by elevated MDA levels, reflecting active lipid peroxidation and oxidative membrane damage [15]; increased SOD activity relative to controls, consistent with a compensatory response to excessive superoxide generation that remains insufficient to restore redox homeostasis [15,24]; decreased GPx activity, indicating impaired peroxide detoxification and collapse of downstream antioxidant defense mechanisms [15,22]; and reduced total tear protein levels, reflecting loss or oxidative modification of protective tear components and overall impairment of tear film integrity in an oxidative environment [15,27].

This distinctive profile—high MDA, elevated SOD, reduced GPx, and decreased tear proteins—describes an ocular surface exposed to persistent oxidative stress in the context of an ineffective antioxidant response [15]. Rather than mounting a coordinated adaptive defense, the ocular surface in DED exhibits a dysregulated redox response, in which superoxide is partially dismutated by SOD, while insufficient peroxidase activity allows secondary reactive species to accumulate, resulting in sustained oxidative injury to lipids and proteins. Such conditions are likely to promote chronic inflammation and apoptotic processes at the ocular surface, thereby contributing to the self-perpetuating cycle of dry eye pathology [11,24].

These findings reinforce the concept that targeting redox imbalance—either by enhancing antioxidant capacity or by reducing oxidative burden—may represent a rational strategy for interrupting this pathogenic cycle and improving ocular surface function in DED. Moreover, the comparative analysis of primary and sarcoidosis-associated DED underscores the importance of considering both local and systemic contributors to ocular surface redox homeostasis [14,25]. Placed within the broader context of existing research, these observations highlight oxidative stress as a unifying mechanistic framework for tear film instability, epithelial damage, and inflammation in dry eye disease, with potential implications for future diagnostic and therapeutic approaches [15,31].

### 3.8. Study Limitations

Several limitations should be considered when interpreting these findings. The cross-sectional design limits the ability to infer causal relationships between oxidative stress alterations and tear film dysfunction. The relatively small sample size reflects the low prevalence of this condition and may limit the generalizability of the results. Furthermore, both eyes were included in the analysis for BUT and Schirmer measurements. Although inter-eye comparisons did not demonstrate statistically significant differences, the inclusion of both eyes may not fully account for within-subject correlation and could introduce a degree of clustering bias. Future studies may benefit from using a predefined single-eye selection strategy or statistical models that explicitly account for inter-eye dependency. Oxidative stress assessment was based on tear-derived biomarkers, which provide indirect insight into ocular surface redox status and may not fully capture tissue-level oxidative processes. Additionally, the lack of simultaneous evaluation of inflammatory mediators precludes direct analysis of the interaction between oxidative stress and inflammation in the pathophysiology of dry eye disease. Although Schirmer testing may induce reflex tearing and potentially influence tear biomarker concentrations, this effect was minimized by the sampling protocol and considered when interpreting the results.

## 4. Materials and Methods

### 4.1. Ethical Approval and Study Protocol

This study was conducted in accordance with the Declaration of Helsinki for research involving human subjects. Ethical approval was obtained from the Ethics Committee of the “Grigore T. Popa” University of Medicine and Pharmacy, Iași, Romania (Approval No. 446, 28 May 2024). All participants provided written informed consent and were informed about data confidentiality and the non-invasive nature of the procedures.

### 4.2. Study Population and Selection Criteria

A total of 60 eyes from 30 participants were included, divided equally into three groups (*n* = 10 patients per group). The primary group consisted of patients with a confirmed systemic diagnosis of sarcoidosis based on clinical and histopathological criteria. The comparative group included patients with primary dry eye disease, while the control group consisted of healthy subjects without ocular pathology.

Inclusion criteria required confirmed sarcoidosis for the primary group and the presence of clinical signs of dry eye for the comparative group. Exclusion criteria included recent ocular surgery, active anterior segment infection, contact lens use within 24 h, or the use of topical medications that could alter tear composition.

### 4.3. Tear Collection Procedure and Rationale

Bilateral tear sampling was performed for each participant using complementary methods for clinical assessment and biochemical analysis. All samples were collected on the same day in a controlled environment (23 °C, 45% relative humidity) to reduce intersession variability. Participants spent 20 min acclimating to the collection room before sampling, during which they read and signed the informed consent, allowing basal tear secretion to stabilize.

Collection started with the right eye using the microcapillary technique. A 10 μL micropipette tip was used to apply gentle suction and collect 1–3 μL of tear fluid from the external canthus, avoiding contact with the cornea or eyelid margins to prevent reflex tearing. After completing the right eye collection, sampling of the left eye was performed after a 30 min interval to minimize the effects of local stimulation or contralateral reflex tearing.

The Schirmer I test without topical anesthesia was then performed on the left eye. A graduated filter paper strip was placed in the inferior conjunctival fornix for 5 min, and wetting was measured to quantify tear volume and support clinical evaluation. The asymmetric use of collection methods and fixed order of procedures preserved the biochemical integrity of samples intended for oxidative stress analysis.

### 4.4. Sample Storage and Preservation

Immediately after collection, all tear samples were transferred into sterile, labeled Eppendorf tubes. To halt enzymatic activity and prevent lipid peroxidation, samples were frozen at −50 °C until further analysis.

### 4.5. Sample Preparation and Bioanalytical Procedures

Frozen samples were gradually thawed at 4 °C to maintain enzymatic stability. Repeated freeze–thaw cycles were avoided. Samples were diluted 1:10 (*w*/*v*) in cold phosphate-buffered saline (PBS) and homogenized. Redox profiling was performed using a high-precision spectrophotometer (Specord 210 Plus, Analytik Jena, Jena, Germany) with quartz cuvettes. Enzymatic activities were normalized to total soluble protein (TSP) to allow accurate intergroup comparisons by accounting for inter-sample variability in tear volume and protein content.

### 4.6. Enzymatic Activity Assays (SOD and GPx)

Total SOD activity was determined using a commercial spectrophotometric assay kit (Sigma-Aldrich, Taufkirchen, Germany), based on inhibition of the superoxide-dependent reduction in a water-soluble tetrazolium salt catalyzed by xanthine oxidase. After incubation at 37 °C for 20 min, absorbance was measured at 450 nm, and results were expressed as percentage inhibition relative to controls.

GPx activity was assessed by monitoring NADPH consumption at 340 nm (GPx assay kit, Sigma-Aldrich, Taufkirchen, Germany). Enzymatic activity was calculated based on the rate of NADPH oxidation, corrected for sample dilution, and expressed as enzymatic units per milliliter (U/mL).

### 4.7. Lipid Peroxidation (MDA) and Total Protein Quantification

MDA levels, as a marker of oxidative lipid damage, were quantified using the thiobarbituric acid (TBA) reaction. After incubation and centrifugation, absorbance was measured at 532 nm and interpolated against a calibration curve prepared with malondialdehyde bis(diethyl acetal).

Protein concentration was measured using the Bradford assay (Sigma-Aldrich, Taufkirchen, Germany), based on Coomassie Brilliant Blue binding. Absorbance at 595 nm was compared to a bovine serum albumin (BSA) standard curve. Total proteins were expressed as concentration (mg/mL).

#### Statistical Analysis

Data were analyzed using IBM SPSS Statistics (version 26.0, Chicago, IL, USA). Continuous variables are presented as mean ± standard deviation unless otherwise specified. A *p*-value < 0.05 was considered statistically significant.

Normality of data distribution was assessed using the Shapiro–Wilk test, given the small sample size (*n* = 10 per subgroup), with a significance threshold set at *p* > 0.05.

For samples collected by capillary, MDA showed a normal distribution in all groups (Control, S-DED, and DED). Proteins, SOD, and GPx demonstrated non-normal distributions, as at least one group violated the normality assumption. BUT and Schirmer values obtained from capillary samples were normally distributed across all groups.

For samples collected by Schirmer, MDA, Proteins, SOD, BUT, and Schirmer test values exhibited normal distributions in all study groups. In contrast, GPx showed a non-normal distribution, with normality violated in the S-DED and DED groups.

Accordingly, parametric statistical tests were applied only to variables demonstrating normal distribution in all groups, while non-parametric tests were used for variables with non-normal distributions.

To account for paired-eye measurements and the use of different tear collection methods in contralateral eyes, linear mixed-effects models were used for confirmatory analyses. Patient group, tear collection method (capillary vs. Schirmer), and their interaction were included as fixed effects, with patient included as a random intercept. Post hoc comparisons were performed using Bonferroni-adjusted estimated marginal means.

Exploratory between-group comparisons were performed using the Mann–Whitney *U* test, stratified by tear collection method, to describe unadjusted differences between control, S-DED, and DED groups. Effect sizes were calculated for Mann–Whitney comparisons using the formula r = |Z|/√N. Given the exploratory nature of the analyses, *p*-values were interpreted descriptively, and no formal correction for multiple comparisons was applied.

Associations between tear biomarkers and clinical tear function parameters (Schirmer test and BUT) were explored using Spearman’s rank correlation coefficients, performed separately by collection method and patient group. Correlation analyses were considered exploratory.

## 5. Conclusions

Dry eye disease is associated with distinct oxidative stress profiles that extend beyond a uniform increase in reactive oxygen species and reflect specific patterns of antioxidant dysfunction at the tear film level. Simultaneous evaluation of lipid peroxidation, antioxidant enzyme activity, and tear protein content indicates that primary dry eye disease and sarcoidosis-associated dry eye exhibit qualitatively different redox signatures. Primary dry eye disease is characterized predominantly by increased lipid peroxidation, as reflected by elevated tear malondialdehyde levels, whereas sarcoidosis-associated dry eye displays a more profound impairment of antioxidant defense, marked by a dissociation between preserved or increased superoxide dismutase activity and significantly reduced glutathione peroxidase activity.

The identification of this SOD–GPx imbalance highlights a previously underexplored mechanism of oxidative stress in secondary dry eye associated with systemic inflammatory disease, suggesting that ineffective downstream detoxification of peroxides may sustain oxidative injury even in the absence of excessive accumulation of terminal lipid peroxidation products. In parallel, the observed reduction in total tear protein levels underscores the functional impact of oxidative stress on tear film composition and stability, reinforcing the concept that oxidative damage affects not only lipid membranes but also protein components essential for ocular surface homeostasis.

Importantly, the correlations between oxidative stress markers and clinical parameters of tear film dysfunction support the physiological relevance of these biochemical alterations and emphasize the value of tear-based redox biomarkers in characterizing dry eye phenotypes. Together, these findings provide new insight into the heterogeneity of oxidative stress mechanisms in dry eye disease and suggest that sarcoidosis acts as a modifier of ocular surface redox homeostasis. The delineation of distinct oxidative signatures in primary versus sarcoidosis-associated dry eye may have implications for the development of more targeted diagnostic and therapeutic approaches, tailored to the underlying redox imbalance rather than to tear deficiency alone.

These observations support the relevance of tear-based redox profiling as a complementary approach for characterizing dry eye phenotypes, particularly in the context of systemic inflammatory disease.

## Figures and Tables

**Table 1:** Descriptive parameters (Median (Interquartile range)).

Capillary		MDA	Proteins	SOD	GPx	BUT	Schirmer
	Control	0.84 (0.13)	30.48 (10.53)	6.50 (2.96)	353.70 (305.47)	16.22 (2.37)	22.50 (8.50)
	S-DED	0.84 (0.43)	25.08 (4.35)	7.42 (1.42)	64.31 (209.00)	4.63 (4.64)	8.00 (11.50)
	DED	1.24 (0.24)	17.19 (6.71)	7.71 (2.34)	192.93 (289.39)	4.87 (4.61)	7.50 (4.00)
Schirmer		MDA	Proteins	SOD	GPx	BUT	Schirmer
	Control	0.92 (0.18)	48.37 (23.62)	3.68 (1.69)	289.39 (353.70)	16.18 (2.85)	21.00 (9.25)
	S-DED	1.15 (0.54)	38.11 (15.46)	4.85 (2.63)	64.31 (64.31)	4.28 (4.44)	9.00 (16.50)
	DED	1.64 (0.54)	31.40 (22.64)	7.14 (6.53)	96.46 (273.31)	3.90 (5.18)	6.00 (5.75)

**Table 2:** Estimated marginal means (Mean ± SE).

	MDA	Proteins	SOD	GPx
Control *	0.89 ± 0.05	38.74 ± 2.2	5.97 ± 0.86	337.62 ± 40.11
S-DED *	1.06 ± 0.05	30.96 ± 2.2	7.29 ± 0.86	125.40 ± 40.11
DED *	1.48 ± 0.05	25.11 ± 2.2	7.07 ± 0.86	218.65 ± 40.11
Capillary **	1.01 ± 0.04	22.55 ± 1.75	8.36 ± 0.71	261.52 ± 32.75
Schirmer **	1.28 ± 0.04	40.65 ± 1.75	5.20 ± 0.71	192.93 ± 32.75

* Analyzed groups averaged over capillary and Schirmer collection methods. ** Collection methods averaged over analyzed groups.

**Table 3:** Pairwise comparisons (Mean ± SE).

Capillary vs. Schirmer				
	MDA	Proteins	SOD	GPx
Control	−0.07 ± 0.07	−23.95 ± 3.55	4.79 ± 1.56	70.74 ± 66.58
	*p* = 0.331	*p* < 0.001	*p* = 0.005	*p* = 0.297
S-DED	−0.30 ± 0.07	−14.71 ± 3.55	3.60 ± 1.56	57.88 ± 66.58
	*p* < 0.001	*p* < 0.001	*p* = 0.005	*p* = 0.392
DED	−0.46 ± 0.07	−15.63 ± 3.55	1.10 ± 1.56	77.17 ± 66.58
	*p* < 0.001	*p* < 0.001	*p* = 0.005	*p* = 0.257
Between analyzed groups				
	MDA	Proteins	SOD	GPX
Control vs. S-DED	−0.17 ± 0.07	7.78 ± 3.02	−1.32 ± 1.21	212.22 ± 56.73
	*p* = 0.076	*p* = 0.039	*p* = 0.839	*p* = 0.001
Control vs. DED	−0.59 ± 0.07	13.63 ± 3.02	−1.10 ± 1.21	118.97 ± 56.73
	*p* < 0.001	*p* < 0.001	*p* = 1.00	*p* = 0.122
S-DED vs. DED	−0.42 ± 0.07	5.86 ± 3.02	0.22 ± 1.21	−93.25 ± 56.73
	*p* < 0.001	*p* = 0.174	*p* = 1.00	*p* = 0.318
Between collection methods				
	MDA	Proteins	SOD	GPX
Capillary vs. Schirmer	−0.27 ± 0.06	−18.10 ± 2.47	3.16 ± 0.99	68.60 ± 46.32
	*p* < 0.001	*p* < 0.001	*p* = 0.002	*p* = 0.144

**Table 4:** Adjusted means for each group (Mean ± SE).

		MDA	Proteins	SOD	GPx
Control	Capillary	0.86 ± 0.07	26.76 ± 3.02	8.37 ± 1.21	372.99 ± 56.73
	Schirmer	0.93 ± 0.07	50.71 ± 3.02	3.58 ± 1.21	300.25 ± 56.73
S-DED	Capillary	0.91 ± 0.07	23.61 ± 3.02	9.10 ± 1.21	154.34 ± 56.73
	Schirmer	1.21 ± 0.07	38.32 ± 3.02	5.50 ± 1.21	96.46 ± 56.73
DED	Capillary	1.25 ± 0.07	17.29 ± 3.02	7.62 ± 1.21	257.24 ± 56.73
	Schirmer	1.71 ± 0.07	32.92 ± 3.02	6.52 ± 1.21	180.06 ± 56.73

**Table 5:** Mann–Whitney *U* test results with effect size.

	BUT	Schirmer
Control vs. S-DED	U = 23	U = 57
	*p* < 0.001	*p* < 0.001
	r = 0.76	r = 0.61
Control vs. DED	U = 0	U = 0
	*p* < 0.001	*p* < 0.001
	r = 0.86	r = 0.86
S-DED vs. DED	U = 181	U = 170.5
	*p* = 0.607	*p* = 0.423
	r = 0.08	r = 0.13

r = effect size.

## Data Availability

The datasets generated and/or analyzed during the current study are not publicly available but are available from the corresponding author on reasonable request.

## Abbreviations

The following abbreviations are used in this manuscript:

DED	dry eye disease
S-DED	sarcoidosis-associated dry eye disease
MDA	malondialdehyde
SOD	superoxide dismutase
GPx	glutathione peroxidase
ROS	reactive oxygen species
Nrf2	Nuclear factor erythroid 2-related factor 2
BUT	breakup time
PBS	phosphate-buffered saline
TSP	total soluble protein
NADPH	Nicotinamide Adenine Dinucleotide Phosphate
TBA	thiobarbituric acid
BSA	bovine serum albumin
CSCR	serous chorioretinopathy
